# Clinical Profiles and Risk Factors of Acute Myocardial Infarction in Young Adults: A Cross-Sectional Study in Myanmar

**DOI:** 10.7759/cureus.71690

**Published:** 2024-10-17

**Authors:** Kyaw L Tun, Zin M Phyu, Nyein Thuzar Tint, Zin H Naung, Thit H Aung

**Affiliations:** 1 Department of Medicine, University of Medicine 1 Yangon, Yangon, MMR; 2 Department of Ophthalmology, University of Medicine 1 Yangon, Yangon, MMR; 3 Department of Cardiology, Yangon General Hospital, Yangon, MMR; 4 Department of Medicine, Yangon General Hospital, Yangon, MMR

**Keywords:** acute myocardial infarction, cad: coronary artery disease, cardiology research, clinical profile, myanmar, premature coronary artery disease, risk factors, young adults

## Abstract

Introduction

Coronary artery disease (CAD) remains a leading cause of morbidity and mortality globally, with an increasing prevalence of acute myocardial infarction (AMI) among younger populations. Despite this rising trend, there are limited data from Myanmar on the clinical profile and associated risk factors for premature CAD in young adults. This study aims to investigate the clinical characteristics and predisposing risk factors for AMI in individuals aged 40 years and below, contributing to a better understanding of disease patterns in this population.

Methods

A cross-sectional descriptive study was conducted at the coronary care unit of Yangon General Hospital over a 12-month period from January 1, 2019, to December 31, 2019. A total of 59 young adults, diagnosed with AMI based on the Fourth Universal Definition of Myocardial Infarction, were included. Clinical data, laboratory investigations, and demographic characteristics were collected and analyzed.

Results

Among the 59 participants, 46 (78%) were male, and smoking was prevalent in 45 (76.3%) cases. Dyslipidemia was common, with 46 (77.9%) exhibiting low high-density lipoprotein (HDL) cholesterol levels and 27 (45.8%) having elevated total cholesterol. Hypertension was observed in 31 (52.5%) patients, and 32 (54.2%) reported a family history of premature atherosclerotic cardiovascular disease. Furthermore, 28 (47.5%) of the cohort were classified as overweight, and 26 (44.1%) demonstrated low levels of physical activity. Chest pain was universally reported by all 59 (100%) patients as the presenting symptom. ST-segment elevation myocardial infarction (STEMI) was the predominant type, affecting 47 (79.6%) patients, with anterior wall involvement in 36 (61%) cases.

Conclusion

The findings of this study reveal that AMI in young adults is more prevalent among males, with smoking and dyslipidemia being the most significant risk factors. The high prevalence of low physical activity, hypertension, and overweight status further underscores the need for early lifestyle interventions. These results can be directly applied to clinical practice and public health policy in Myanmar by prioritizing smoking cessation programs, improving dyslipidemia management, and promoting physical activity in young populations. Additionally, this study provides a foundation for further research to explore more specific risk factors and paves the way for broader studies focusing on young AMI cases in Myanmar.

## Introduction

Acute myocardial infarction (AMI), a common presentation of coronary heart disease, occurs when blood flow decreases or stops in a part of the heart, causing damage to the heart muscle. The World Health Organization estimated that 12.2% of worldwide deaths are caused by ischemic heart disease, making it the leading cause of death in high- and middle-income countries and second only to lower respiratory tract infections in lower-income countries [1]. Globally, more than three million people experience ST-segment elevation myocardial infarction (STEMI), and four million experience non-ST-segment elevation myocardial infarction (NSTEMI) each year [2].

Ischemic heart disease is becoming a more common cause of death in developing countries, with a rising trend and high mortality observed in Myanmar [3]. Disability-adjusted life years (DALYs) lost to ischemic heart disease are expected to account for 5.5% of the total by 2030, making it one of the most significant causes of disability and death [1].

In South Asia, the median age for the first presentation of AMI is 53 years, compared to 63 years in Western Europe and China. AMI occurs earlier in men than in women, with the first attack recorded in 4.4% of Asian women and 9.7% of Asian men under the age of 40, rates twofold to threefold higher than those observed in Western populations [4]. Although AMI is less common in individuals under the age of 40, there is an increasing number of young patients being affected [5]. This age group is particularly significant because coronary artery disease in young adults can lead to devastating consequences for patients, their families, and society [6].

Preventing coronary artery diseases in younger people is crucial and can be more cost-effective than treating established diseases. Thus, it is essential to understand the risk factors for premature AMI [7]. Much of the knowledge regarding the clinical presentation and risk factors for premature AMI has been acquired from studies conducted in other populations. However, the relevance of these findings to the Myanmar population remains unclear. This descriptive study was conducted to characterize the presentation and risk factors for premature AMI in patients admitted to Yangon General Hospital, Myanmar, to help develop strategies to prevent AMI in young patients in Myanmar.

## Materials and methods

This hospital-based, prospective cross-sectional descriptive study was conducted at the Department of Cardiology, Yangon General Hospital, over a one-year period from January 2019 to 31 December 2019, focusing on young patients aged 40 years or younger diagnosed with AMI. Yangon General Hospital was selected as it is the largest tertiary care center in Myanmar and a national referral center for cardiovascular diseases (CVDs), including AMI.

The study was conducted according to the ethical guidelines established by the Research and Ethics Committee of the University of Medicine 1, Yangon. The research proposal was submitted and approved (reference number: Med-0027/2018). All participants were invited to take part voluntarily, and the study objectives, procedures, benefits, and risks were clearly explained. Informed consent was obtained from each participant prior to their inclusion in the study. Strict confidentiality was maintained through the use of coded data, and participants had the right to withdraw at any time without any consequences. No incentives or additional charges were associated with participation.

The sample size was calculated using the sample size determination formula for proportions by Daniel and Cross (2013) [8], with a 95% confidence level, an estimated 70% proportion, and a 14% margin of error, yielding a required minimal sample size of 42. Sixty-six young AMI cases were admitted to the Cardiology Department of Yangon General Hospital in 2018.

$n = \frac{z_{(1-\alpha/2)}^2 \cdot p(1-p)}{d^2}$

**Figure 1:**
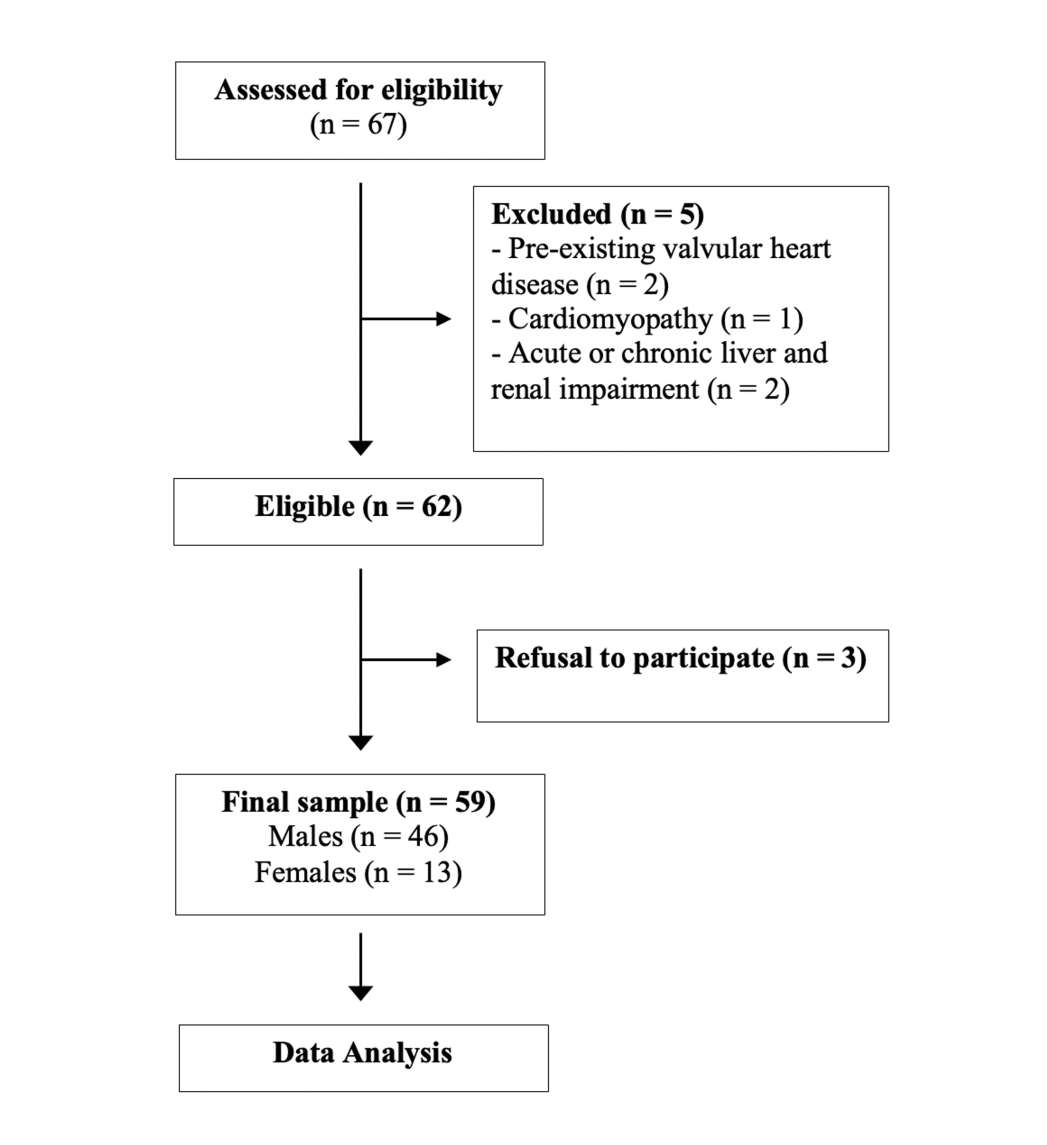
STROBE diagram for study on acute myocardial infarction in young adults STROBE = Strengthening the Reporting of Observational Studies in Epidemiology

Patients were selected using consecutive sampling, which involved including all eligible young AMI patients admitted to the coronary care unit during the study period. The inclusion criteria were patients aged between 18 and 40 years with confirmed AMI, according to the Fourth Universal Definition of Acute Myocardial Infarction (2018) [9]. Exclusion criteria included pre-existing valvular heart disease, cardiomyopathy, or acute or chronic liver impairment (defined as ALT >36 IU/L and bilirubin >17 µmol/L) and renal impairment (defined as creatinine levels >106 µmol/L for males and >96 µmol/L for females), as these conditions can mimic AMI and lead to elevated troponin T levels. During the study period, 67 young AMI patients were admitted to the acute coronary care unit of Yangon General Hospital. The patients were assessed for eligibility; five were excluded due to pre-existing conditions, and three declined participation. The final sample size was 59, as outlined in Figure 1.

Data collection involved detailed clinical history-taking, including presenting symptoms, smoking habits, alcohol consumption, hypertension, diabetes, physical activity status, and family history of premature atherosclerotic cardiovascular disease (ASCVD) using a standard questionnaire. Physical examinations, including body mass index (BMI), blood pressure, and waist circumference, were recorded using standardized equipment by trained data collectors. We assessed both inter-observer and intra-observer variability for measurements to ensure consistency and reliability.

Laboratory analyses for full blood count, liver function, renal function, fasting lipid profile, fasting homocysteine levels, hemoglobin A1c, and random blood glucose were performed using the Roche cobas c311 analyzer (Roche Diagnostics Corporation, Indianapolis, IN), Sysmex XN-1000 hematology analyzer (Sysmex Corporation, Kobe, Japan), and standardized Roche Accu-chek® Inform II system glucometer (Roche Diagnostics Corporation, Indianapolis, IN). Standardized Nihon Kohden CardioFax C ECG-1150 (Nihon Kohden, Tokyo, Japan) was used to determine the site of infarction and ST-segment abnormalities. The equipment used in this study underwent regular calibration and quality control measures to ensure accuracy in laboratory analyses.

Hypertension was defined as a systolic blood pressure >140 mmHg and/or a diastolic blood pressure >90 mmHg and/or the use of antihypertensive drugs. Diabetes mellitus was defined as fasting plasma glucose ≥7.0 mmol/L, random plasma glucose ≥11.0 mmol/L, hemoglobin A1c (HbA1c) ≥6.5%, or a previous diagnosis of DM. Dyslipidemia was defined as total plasma cholesterol >200 mg/dL; low-density lipoprotein (LDL) >130 mg/dL; triglycerides >150 mg/dL; high-density lipoprotein (HDL) ≤40 mg/dL, or the use of cholesterol-lowering drugs. Polycythemia was defined as a serum hematocrit >49% in men and >48% in women, or hemoglobin >16.5 g/dL in men or >16.0 g/dL in women. Hyperhomocysteinemia was defined as a plasma homocysteine level above 15 µmol/L. Family history of premature ASCVD (<55 years old in men, <65 years old in women) was defined as any myocardial infarction or stroke in a first-degree relative. Patients who currently smoked or stopped smoking less than a year ago were defined as smokers, while all others were defined as nonsmokers. Patients who drank more than 14 units of alcohol per week were identified as having unhealthy alcohol use. Overweight was defined by a BMI ≥25 kg/m². Central obesity was defined as a waist circumference >94 cm in men and >80 cm in women. Information about illicit drug use was obtained. Physical activity levels were assessed using the International Physical Activity Questionnaire (IPAQ) in the Myanmar language [10].

Statistical analysis was conducted using Statistical Package for the Social Sciences (SPSS) software version 16.0 (SPSS for Windows, SPSS Inc., Chicago, IL). Descriptive statistics were used to analyze the findings. Categorical variables, such as demographic features and risk factors, including age group, sex distribution, physical activity, smoking, and alcohol consumption, were presented as frequencies and percentages. Continuous variables, such as biometric and biochemical measurements, were categorized as normal or abnormal according to the operational definitions and then described as frequencies and percentages. All risk factors were presented by gender distribution.

## Results

A total of 59 young patients with AMI participated in this study, including 46 males (78.0%) and 13 females (22.0%). The majority of participants were aged between 35 and 40 years (n = 35, 59.3%), while the second-largest age group was 30 to 34 years (n = 20, 33.9%).

Significant gender disparities were observed in cardiovascular risk factors. Smoking was reported by 43 males (93.5%) and two females (15.4%), while heavy alcohol consumption was noted in 31 males (67.4%) compared to one female (7.7%). Hypertension was diagnosed in 21 males (45.7%) and 10 females (76.9%), and diabetes mellitus affected six females (46.2%) and seven males (15.2%). A family history of premature CVD was present in 23 males (50.0%) and nine females (69.2%). Additionally, females exhibited higher rates of central obesity (n = 10, 76.9%) and overweight (n = 11, 84.6%) compared to males, where central obesity was found in 15 males (32.6%) and overweight in 17 males (37.0%). Low physical activity was more common among females (n = 8, 61.5%) compared to males (n = 18, 39.1%).

Dyslipidemia was prevalent across both genders. Elevated cholesterol levels (>200 mg/dL) were found in 19 males (41.3%) and eight females (61.5%). Low HDL cholesterol (<40 mg/dL) was more common in males (n = 38, 82.6%) than in females (n = 8, 61.5%), while elevated LDL cholesterol (>130 mg/dL) affected 20 males (43.5%) and seven females (53.9%). Elevated triglycerides (>150 mg/dL) were present in 15 males (32.6%) and eight females (61.5%). Polycythemia was identified in nine males (19.6%), with no cases among females.

Cocaine use was reported in three males (6.5%) and none of the females. Hyperhomocysteinemia was found in four males (8.7%) and none of the females (Table 1).

**Table 1:** Age, gender, and risk factor profiles for cardiovascular disease in the study population ASCVD = atherosclerotic cardiovascular disease; HDL = high-density lipoprotein; LDL = low-density lipoprotein Polycythemia = serum hematocrit >49% in men and >48% in women, or hemoglobin >16.5 g/dL in men or >16.0 g/dL in women.

Category	Male (n = 46)	Female (n = 13)	Total (n = 59)
Age group (years)			
18-25	1 (2.2%)	0 (0.0%)	1 (1.7%)
25-29	3 (6.5%)	0 (0.0%)	3 (5.1%)
30-34	16 (34.8%)	4 (30.8%)	20 (33.9%)
35-40	26 (56.5%)	9 (69.2%)	35 (59.3%)
Risk factor			
Smoking	43 (93.5%)	2 (15.4%)	45 (76.3%)
Alcohol use	31 (67.4%)	1 (7.7%)	32 (54.2%)
Hypertension	21 (45.7%)	10 (76.9%)	31 (52.5%)
Diabetes mellitus	7 (15.2%)	6 (46.2%)	13 (22.0%)
Family history of premature ASCVD	23 (50.0%)	9 (69.2%)	32 (54.2%)
Central obesity	15 (32.6%)	10 (76.9%)	25 (42.4%)
Overweight	17 (37.0%)	11 (84.6%)	28 (47.5%)
Low physical activity	18 (39.1%)	8 (61.5%)	26 (44.1%)
Polycythemia	9 (19.6%)	0 (0.0%)	9 (15.3%)
Cholesterol >200 mg/dL	19 (41.3%)	8 (61.5%)	27 (45.8%)
HDL <40 mg/dL	38 (82.6%)	8 (61.5%)	46 (77.9%)
LDL >130 mg/dL	20 (43.5%)	7 (53.9%)	27 (45.8%)
Triglycerides >150 mg/dL	15 (32.6%)	8 (61.5%)	23 (39.0%)
Cocaine use	3 (6.5%)	0 (0.0%)	3 (5.1%)
Hyperhomocysteinemia	4 (8.7%)	0 (0.0%)	4 (6.8%)

Chest pain was the predominant presenting symptom, reported by all 59 patients (100%). Other common symptoms included perspiration in 48 patients (81.4%), breathlessness in 33 patients (55.9%), and palpitations in 31 patients (52.5%). Vomiting occurred in 25 patients (42.4%), while syncope was noted in nine patients (15.3%). Among the most frequent signs were tachypnea in 16 patients (27.1%), tachycardia in 12 patients (20.3%), and elevated blood pressure in 17 patients (28.8%). Bradycardia and low blood pressure were each observed in three patients (5.1%). Cyanosis was rare, affecting only one patient (1.7%). Most patients were classified as Killip class I (52 patients, 88.1%), while five patients (8.5%) were in class II, and two patients (3.4%) were in class III; no patients were classified as class IV.

ECG findings revealed that 47 patients (79.6%) had ST-elevation myocardial infarction (STEMI), while 12 patients (20.3%) had non-ST-elevation myocardial infarction (NSTEMI). The most common infarction sites were anteroseptal in 22 patients (37.3%) and anterolateral in 14 patients (23.7%), followed by inferior in 12 patients (20.3%), inferior with posterior involvement in eight patients (13.6%), and inferior with right ventricular involvement in three patients (5.1%) (Table 2). There were no significant differences in risk factors or clinical presentation between the STEMI and NSTEMI groups in our cohort. No mortality was observed in this study population.

**Table 2:** Clinical presentation and ECG findings in the study population *Kilip classification: Killip class I - no clinical signs of heart failure Killip class II - rales or crackles in the lungs, a third heart sound, and elevated jugular venous pressure Killip class III - frank acute pulmonary edema Killip class IV - cardiogenic shock or hypotension (measured as systolic blood pressure lower than 90 mmHg) and evidence of peripheral vasoconstriction (oliguria, cyanosis, or sweating)

Category	Number (percentage)
Presenting symptoms	
Chest pain	59 (100%)
Perspiration	48 (81.36%)
Breathlessness	33 (55.9%)
Palpitation	31 (52.5%)
Vomiting	25 (42.4%)
Syncope	9 (15.3%)
Presenting signs	
Tachycardia	12 (20.3%)
Bradycardia	3 (5.1%)
Tachypnea	16 (27.1%)
Elevated blood pressure	17 (28.8%)
Low blood pressure	3 (5.1%)
Cyanosis	1 (1.7%)
Killip classification*	
Class I	52 (88.1%)
Class II	5 (8.5%)
Class III	2 (3.4%)
Class IV	0 (0.0%)
ECG changes	
ST-elevation myocardial infarction	47 (79.6%)
Non-ST-elevation myocardial infarction	12 (20.3%)
Site of infarction in ECG	
Anteroseptal	22 (37.3%)
Anterolateral	14 (23.7%)
Inferior	12 (20.3%)
Inferior + posterior	8 (13.6%)
Inferior + right ventricular	3 (5.1%)

## Discussion

This study was designed to identify the clinical profile and risk factors of young patients with AMI in Myanmar, where no similar studies had previously been conducted. It provides an essential contribution to the understanding of AMI in our young adult population. 

In our study, 35 patients (59.3%) were aged between 35 and 40 years, and only four patients (6.8%) were under 30 years old. The mean age of the patients was 35 years (SD = 4.05), with the youngest being 22 years and the oldest 40 years. The prevalence of AMI was higher in males (46, 78.0%) compared to females (13, 22.0%). This finding aligns with a previous study by Lyengar et al. [11] conducted in India. The lower prevalence of AMI in young females may be attributed to the protective effects of estrogen in pre-menopausal women.

Smoking was strongly associated with premature AMI, affecting 45 patients (76.3%). This finding is consistent with other studies identifying smoking as a major risk factor for coronary artery disease in young individuals [4,12]. Additionally, 28 patients (47.5%) reported unhealthy alcohol consumption, aligning with findings from the INTERHEART Study [4].

A family history of premature ASCVD was reported by 32 patients (54.2%), with the majority having a history of first-degree relatives. This highlights the importance of genetic factors in the development of premature ischemic heart disease. This finding is consistent with a previous study by Chacko et al. [13].

Evaluation of conventional risk factors revealed that 31 patients (52.5%) had hypertension, 13 patients (22.0%) had diabetes, 27 patients (45.8%) had elevated cholesterol levels, 27 patients (45.8%) had increased LDL cholesterol, 46 patients (77.9%) had reduced HDL cholesterol, and 23 patients (39.0%) had elevated triglycerides. The prevalence of these risk factors in our study was higher compared to previous studies [11,12]. These findings highlight dyslipidemia as a significant modifiable risk factor in young AMI patients. In contrast, our study noted a higher percentage of young AMI patients in Myanmar with reduced HDL cholesterol. The high smoking rates, high BMI, and low physical activity observed in the study likely contribute to these lipid abnormalities. Furthermore, the limited healthcare infrastructure in Myanmar may also contribute to a lack of early screening and management of lipid disorders, exacerbating the issue. Genetic factors in Myanmar populations could further explain the higher rates of reduced HDL cholesterol, although more research is needed to clarify these links.

Our study revealed that 26 patients (44.1%) had low physical activity. This finding is consistent with previous studies that indicate reduced physical activity as a risk factor for coronary artery disease [4,11]. In terms of BMI, 28 patients (47.5%) were overweight, and central obesity, indicated by increased waist circumference, was observed in 25 patients (42.4%). These results are similar to those found in previous studies on risk factors [4,11].

Polycythemia was observed in nine patients (15.3%), all of whom were active smokers with no prior thrombotic episodes. The strong association between smoking and polycythemia in this study is consistent with secondary polycythemia, which results from chronic hypoxia induced by smoking. While case reports suggest a possible link between polycythemia rubra vera and AMI, it is more likely that the elevated hematocrit levels seen in our patients are due to smoking, increasing cardiovascular risk.

Hyperhomocysteinemia was found in four patients (6.8%). This contrasts with a previous study focusing on hyperhomocysteinemia in a young AMI population by Sun et al. [14]. Our findings show that hyperhomocysteinemia as a vascular thrombotic risk factor is not common in young AMI patients in Myanmar.

Three participants (5.1%) were cocaine users. These patients did not have significant traditional risk factors, and their angiogram results showed normal coronary arteries, suggesting that coronary vasospasm might be the cause, as previously discussed by Rezkalla et al. [15]. Although the number is small, this issue requires further attention and public intervention.

Chest pain was the predominant symptom, reported by all 59 patients (100%), followed by perspiration in 48 patients (81.4%), breathlessness in 33 patients (55.9%), palpitations in 31 patients (52.5%), vomiting in 25 patients (42.4%), and syncope in nine patients (15.3%). The most common signs included elevated blood pressure in 17 patients (28.8%), tachypnea in 16 patients (27.1%), tachycardia in 12 patients (20.3%), low blood pressure in three patients (5.1%), bradycardia in three patients (5.1%), and cyanosis in one patient (1.7%). The majority of patients were classified as Killip class I (52 patients, 88.1%), with five patients (8.5%) in class II and two patients (3.4%) in class III. No mortality was observed during the hospitalization period. These findings are consistent with a previous study on young AMI patients, which also found chest pain and perspiration to be the most common symptoms [12].

In our study, ST-segment elevation AMI was more common, affecting 47 patients (79.6%), while non-ST-segment elevation AMI occurred in 12 patients (20.3%). The most frequent sites of infarction were anteroseptal in 22 patients (37.3%), anterolateral in 14 patients (23.7%), inferior in 12 patients (20.3%), inferior with posterior wall extension in eight patients (13.6%), and inferior with right ventricular involvement in three patients (5.1%). ST-segment elevation involving the anterior wall was the most common presentation in Myanmar young adults with AMI. Similar trends have been observed in previous studies [12].

As a key highlight, the high prevalence of smoking, dyslipidemia, and family history of premature CVD among young AMI patients in Myanmar calls for urgent public health interventions. Smoking cessation programs, along with initiatives to promote physical activity and improve lipid management, are essential. Early screening for cardiovascular risk factors in families with a history of ASCVD could help reduce future cases of premature AMI.

This study fills an important gap in the literature on AMI in young adults in Myanmar, providing crucial data that can improve both public health strategies and clinical practice. While other studies have explored AMI in young adults globally, our findings highlight specific patterns in Myanmar, such as the particularly high prevalence of smoking and reduced HDL cholesterol, which require tailored approaches to prevention and treatment.

Limitations

Our study has several important limitations. First, although it was conducted at the largest tertiary cardiology center in Myanmar, it is still a single-center study, and the findings may not be fully generalizable to the broader national population. Additionally, the absence of angiographic data, genetic testing, and long-term follow-up limits our ability to comprehensively assess the full extent of coronary artery disease, hereditary risk factors, and long-term outcomes in young adults with AMI. Furthermore, the cross-sectional, descriptive nature of the study limits our ability to explore the associations between risk factors and AMI in young adults in depth. Despite these limitations, we believe this study provides critical insights into the clinical profiles and risk factors of this unique population, laying a strong foundation for future research and targeted interventions.

Future studies should explore the role of genetic predisposition, as well as the socio-economic and cultural factors influencing high smoking rates and physical inactivity, and assess the impact of public health interventions on managing these risk factors in young AMI patients. Additionally, future studies should adopt a multi-center design with a larger sample size to improve generalizability. Prospective cohort studies would allow for a better understanding of the causality between risk factors and AMI in young adults.

## Conclusions

This study provides the first in-depth analysis of the clinical profile and prevalence of risk factors among young AMI patients in Myanmar. Key findings include a high prevalence of smoking, dyslipidemia with low HDL cholesterol, elevated BMI, low physical activity, and a family history of premature ASCVD, reflecting the unique socio-economic and healthcare challenges in Myanmar. These results underscore the urgent need for targeted prevention strategies to reduce smoking, improve health literacy, and enhance dyslipidemia management. This study fills a crucial gap in understanding AMI in young adults in Myanmar and offers a foundation for future research and public health interventions.
